# The Role of Demography and Markets in Determining Deforestation Rates Near Ranomafana National Park, Madagascar

**DOI:** 10.1371/journal.pone.0005783

**Published:** 2009-06-17

**Authors:** Christopher P. Brooks, Christopher Holmes, Karen Kramer, Barry Barnett, Timothy H. Keitt

**Affiliations:** 1 Department of Biological Sciences, Mississippi State University, Mississippi State, Mississippi, United States of America; 2 Wildlife Conservation Society, International Conservation, Antananarivo, Madagascar; 3 Department of Anthropology, Harvard University, Cambridge, Massachusetts, United States of America; 4 Department of Agricultural Economics, Mississippi State University, Mississippi State, United States of America; 5 Section of Integrative Biology, The University of Texas, Austin, Texas, United States of America; Centre National de la Recherche Scientifique, France

## Abstract

The highland forests of Madagascar are home to some of the world's most unique and diverse flora and fauna and to some of its poorest people. This juxtaposition of poverty and biodiversity is continually reinforced by rapid population growth, which results in increasing pressure on the remaining forest habitat in the highland region, and the biodiversity therein. Here we derive a mathematical expression for the subsistence of households to assess the role of markets and household demography on deforestation near Ranomafana National Park. In villages closest to urban rice markets, households were likely to clear less land than our model predicted, presumably because they were purchasing food at market. This effect was offset by the large number of migrant households who cleared significantly more land between 1989–2003 than did residents throughout the region. Deforestation by migrant households typically occurred after a mean time lag of 9 years. Analyses suggest that while local conservation efforts in Madagascar have been successful at reducing the footprint of individual households, large-scale conservation must rely on policies that can reduce the establishment of new households in remaining forested areas.

## Introduction

The poverty-biodiversity nexus is among the most critical challenges facing global biodiversity conservation [1]. Across the globe, poverty-stricken populations are most likely to have a heavy reliance on locally available natural resources. Not surprisingly, many of the world's biodiversity hotspots occur in regions where local people rely on their natural resources to survive [2]–[3], and where restricted access may deepen the abject poverty under which they already struggle (e.g., [4]). Debates over potential mechanistic links between natural resource protection and poverty in rural communities and how human interests should affect conservation strategies have been contentious [5]. Because the path out of poverty is often made through exploitation of natural resources, it is generally thought that resource conservation will lead to a further deepening of poverty for local people.

In many parts of the tropics, resource use frequently takes the form of subsistence agriculture. Along with timber harvesting [6], the rapid loss of primary forest to subsistence agriculture [7]–[8] ultimately represents one of the greatest threats to global biodiversity. Efforts to conserve biodiversity through the establishment of reserves generally follow one of two models: either a community-based approach, or an exclusionary approach. The former concentrates on balancing the needs of local people with the conservation priorities (e.g., [9]), while the latter involves the establishment of protected reserves that may conflict with the culture or economic well-being of local peoples [10]. Neither strategy has led to widespread success, resulting in debates over the efficacy of both approaches. In reality, many rural, agrarian populations in developing nations may be stuck in a poverty trap [11]. In these populations poverty is self-reinforcing, with a number of potential mechanisms driving the process [12].

This apparently unresolvable conflict between conservation and development [13] may limit the success of locally-oriented conservation strategies. Potential solutions may exist by developing an explicit understanding of how within- and between-village dynamics and market influences [1], [10], [14] affect household-level decisions about deforestation. Rudel [15] suggests that effective conservation of tropical forests will require an understanding of how land tenure, socioeconomic structure, and demography affect local and regional patterns of deforestation. Our goal here is to begin developing this kind of integrated view of the motivations for deforestation in the region near Ranomafana National Park, Madagascar.

Madagascar's highland tropical forests are among the greatest global priorities for the development of effective conservation strategies [16]. The country boasts an exceptionally high proportion of endemic species coupled with rapid human population growth [17]. Of the more than 14,000 endemic species found on this island nation, 90% inhabit the tropical forest remnants of the central highlands [18], including those near Ranomafana. Habitat loss due to deforestation already has caused well-documented losses of Malagasy faunal biodiversity [19]–[22]. Preservation of the remaining forest cover in the highlands is one of the most critical issues in protecting biodiversity in Madagascar. But despite the urgency created by the rapid loss of forest habitat, our understanding of local motivations for deforestation here remain incomplete.

In Madagascar, the reasons for deforestation are as diverse as they are across the tropics. Where subsistence agriculture dominates, there exists a diverse set of agroforestry techniques ranging from slash-and-burn for the cultivation of hillslope rice (tavy plots) to more diverse family fruit gardens (tanimboly) and irrigated rice paddies [23]. Tavy techniques are generally preferred, both for traditional reasons and because they provide a higher yield per unit effort as compared with irrigated paddies (despite having substantially lower yield per hectare). Historically, tavy was a sustainable approach because low population densities allowed for rotation of tavy plots over a 10–20 year cycle. But rapidly growing populations (3% increase per year) and economic depression has led to widespread migration, which ultimately has led to the establishment of numerous new households in rural villages.

Since the seminal work of Chayanov [24] we know that household dynamics, land tenure practices, etc. can have profound influences on the patterns of land use in rural agrarian cultures. Despite the importance of its highland forests, no comprehensive analysis exists to suggest how demography, land tenure, and socioeconomic structure might affect land use patterns in Madagascar, nor how those relationships might change through time. Here we derive a theoretical estimate of the area of land cultivated by individual households. We then describe the demography and land use practices of households in four villages around Ranomafana National Park (RNP) in the context of our model in order to assess the role of markets and demography within and among households and villages. We contend that accurate prediction of future changes to the risk of biodiversity loss due to deforestation in the region will have to explicitly consider the effect of demography in the economic and social structure of villages in the region.

## Materials and Methods

**Figure 1:**
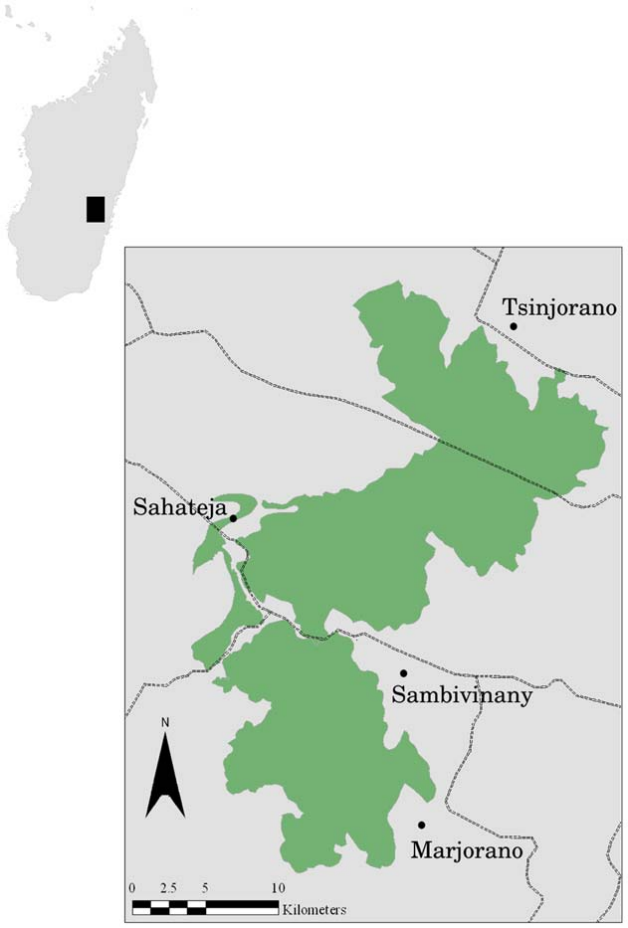
A map of the area around Ranomafana National Park showing the study villages and all roads. The the sole primary road between the RNP region and the major urban centers runs east-west through the center of the park near both Sahateja and Sambivinany.

Ranomafana National Park is a 41,500 ha reserve that lies at the eastern edge of Madagascar's central highland plateau (Fig. 1). The region is home to almost 2,000 endemic species that have been identified to date. Much of the forest that historically covered this region of Madagascar has been cleared, except for the protected land inside the park. Most of this land is used for subsistence agriculture. Rice, the primary staple crop, is grown here in both flooded paddies and along dry hillslopes. Hillslope plots are generally swidden and can produce a viable yield for only one or two seasons, but it is traditionally favored by many of the local people.

Four villages within a 7 km buffer of RNP were chosen to provide a range in the degree of recent immigration. Both Sahateja and Sambivinany likely experience the greatest influence from immigration and have the strongest ties to external markets. Both are located close to the primary road (portions are paved) leading to the two largest urban centers in Madagascar, Antananarivo and Fianarantsoa. In contrast, Marjorano and Tsinjorano are located far from any primary roads. We interviewed heads of households in these four villages and used their oral accounts to determine the location and boundaries of ownership for each plot in these villages. A global positioning system (GPS) was used to establish the boundaries of each of the 1136 plots owned by the 163 households in the study villages. Where aerial photos were available (data from 1991 and 1992), we corrected any small errors in the size and shape of individual plots from field-collected data. We measured the area of forest cleared between 1989 and 2003 in our study villages from oral accounts from heads of households. Any plots that were reported as being newly acquired through clearing of forest were assumed to represent loss of primary forest. The area of each recently cleared plot was calculated using the area of the corresponding polygons in our GIS database.

Demographic information about each of the households in our four study villages was also collected through oral interviews with heads of households. These data include information on the number of individuals in each household, total labor force, including the number who were hired to provide labor in the field (external labor), the migrant status of each household (resident v. migrant) and the number of years the household had been in residence in the current village.

### The Model

In an effort to understand the relative influence of different demographic parameters, both endogenous and exogenous to households, we derived a model for production and consumption that could be used to describe the activities of individual households. The first assumption is that the minimal requirement for any household engaged in agriculture to subsist, absent market influences, is that the supply of food for household members is sufficient to meet their collective physiological demand for food. We further assume that any needs beyond the basal physiological requirements can be estimated as a percentage of total production (ω) in each village. Given these assumptions we can setup the following equality for household *i*

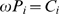
(1)where *P* represents household production and *C* is household-level consumption. We can then model consumption as a linear function of household size (H)
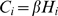
(2)where β is the per-capita consumption rate and the subscript denotes individual households in the village. Household production can then be estimated using a Cobb-Douglass function [25]

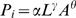
(3)where *L* is the available labor force, *A* is the area currently cultivated, and γ and θ are measures of the elasticity in labor and area. Here we take the total labor force available to a household as a function of household size
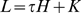
(4)where K is the labor force derived from outside the household and *τ* is the proportion of the household that provides labor. While the application of this model is likely sensitive to the definition of labor (e.g., [26]–[27]) and the time allocation of laborers, we define labor based on the number of individuals reported to assist with agriculture. Plugging each of these expressions into eqn. 1 results in an equation that can be solved for the minimum area that must be cultivated in order for a given household to persist 
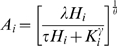
(5)where λ = β/ωα. It is important to note that this represents the minimum requirements for a household completely isolated from market influences. We explicitly developed our model to exclude market influences, not because we expect them to be unimportant, but because we want to separate their effects from household demography. Thus, deviations from our prediction of minimal area cultivated are to be interpreted as the consequence of market influences in our study villages. We expect that ties to markets would imply the ability to supplement production with rice purchases, potentially reducing the household production required to meet household consumption needs. Thus, households which fall below our expectation can exist solely because of their ability to purchase rice in the market. Equation 5 was parameterized using data from the four study villages near RNP using ordinary least-squares regression. Models were fit using the nlminb function in the *R* statistical package[28] to minimize squared deviations from the model.

## Results

Most of the changes in cultivated land were directly related to the clearing of forest and not changes in fallow area. Data suggest a net increase in the total area in fallow across the four villages (from 82.48 km^2^ to 88.91 km^2^) between 2003 and 2004, though the percent change in cultivated area was minimal (∼7%, thus ρ = 0.93). Deforestation rates differed greatly between villages. The most isolated villages (Marjorano and Tsinjorano) had very low rates of deforestation between 1990 and 2004, with the more centrally located villages (Sahateja and Sambivinany) cleared forest at rates two orders of magnitude faster. Of the 1136 plots surveyed, 70 (1.05 km^2^) were cleared between 1990 and 2004, almost all of it by households in Sahateja and Sambivinany. In Sahateja, three migrant households accounted for 95% (0.59 km^2^) of the deforestation. These newly cleared plots in Sahateja were used for paddy rice, while land clearing in Sambivinany was often for tavy, and was more evenly distributed among households.

**Figure 2:**
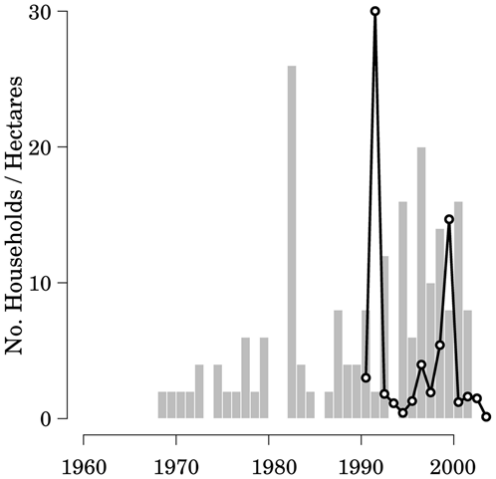
Number of new households (grey bars) in the four study villages (1960–2004) and the total area cleared (black line) (1990–2004). There is an apparent time lag between immigration and area cleared of 9 years that is supported by the mean period between household establishment and initial land clearing (9.19 years). Statistical analysis is detailed in the text.

The annual patterns of immigration and deforestation in the RNP area are shown in Fig. 2. The apparent 9 year time lag between immigration rate and the area deforested in the region (r^2^ = 0.6337, p<0.05) corresponds to the mean time between the establishment of a new household and the year during which land was first cleared (9.19 years). This pattern was indicative of strong differences between resident and immigrant households that were clearly important to the demand for land within villages. The proportion of immigrant households and the mean size of households co-varied across villages. Immigrant households were significantly larger (p = 0.019) and cultivated a larger areas (p = 0.004) than did resident households. Sahateja was the most affected by immigration with 84% of the households in this village immigrating from urban centers or other villages. The mean size of households in Sahateja (7.19 individuals) is much larger than in Tsinjorano (4.42) where only 7% of the households were immigrant.

**Figure 3:**
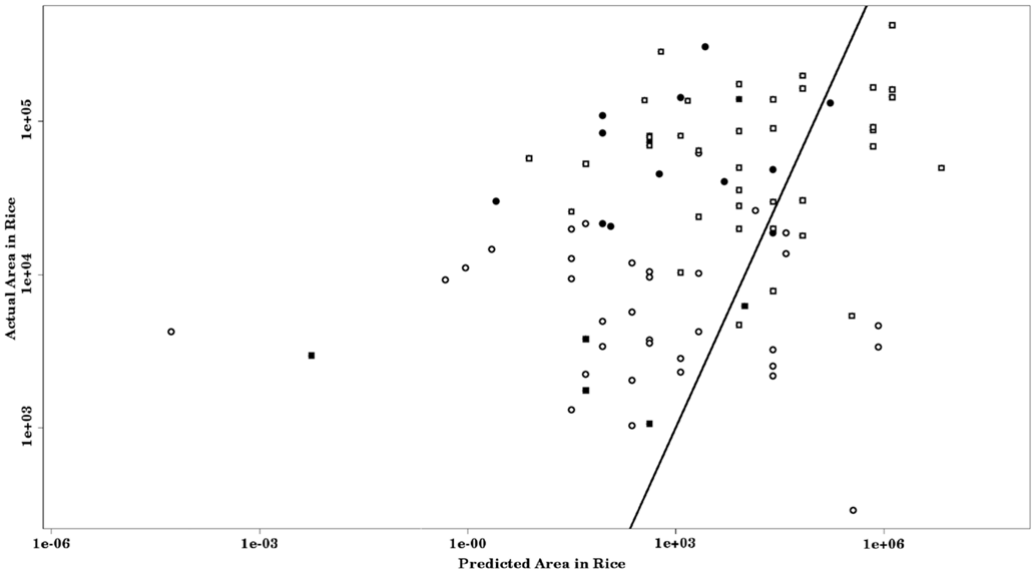
Actual area cultivated in rice plotted versus the predicted minimal area cultivated (from eqn. 5). Open symbols indicate households in villages closer to large urban markets via road (Sambivinany – circle, Sahateja – square). Closed symbols are from villages where households are more likely to be isolated from urban markets (Marjorano – circle, Tsinjorano – square). The line indicates where actual area cultivated is equal to predicted area cultivated.

The estimated minimum conditions for subsistence in households (eqn. 5) suggests that between 63% and 87% of households (global mean of 71%) have more land in agriculture than the minimum amount necessary for subsistence (Table 1, Fig. 3). More than half of the households which cleared less land than predicted are migrant, with ∼89% of them occurring in Sahateja and Sambivinany. While there were not differences in the proportion of yield taken to market in households that sold at least some rice, households which cultivated more land than expected took 36.24 kg of rice to market each year compared with only 11.91 kg for households that cultivated less land than predicted. In addition, only 25.93% of households which farmed less than the predicted land area took rice to market, compared with 44.78% of households which were producing more.

**Table 1:** Parameter estimates for the production-consumption model of deforestation.

Parameter	Estimated Value	Standard Error
α	183.42	0.653
β	27.63	1.789
τ	0.36	0.009
γ	0.39	0.233
ω	0.47	0.013
θ	1×10^−50^	0.063

## Discussion

Analysis suggests that the deforestation in the RNP region over the past 15 years is closely tied to migration and the establishment of new households. This is apparent from the lagged correlation between the establishment of new households and area cleared each year (Fig. 2), and from the correspondence between migrant households and the likelihood of cultivating less land than predicted. Together our data suggest that migrant households that are more likely to be tied to the market and to clear forest. The consequence is that the addition of households into a village has a greater effect on deforestation pressure than do the dynamics within households. This suggests that efforts to conserve the remaining forest along the highland plateau should include measures to reduce the flow of migrants among villages.

The influence of adding households to local villages has been previously reported to have important implications for conservation. Changing demographics within local villages can have profound effects on the patterns of local land use [29]–[30] irrespective of agricultural efficiency. The impact of changing the number of households is likely much more important to understanding biodiversity risks than are changes in household size (though the two are clearly not independent of one another), especially in many of the world's biodiversity hotspots [31]. The strong correlation between the number of new households and area cleared over the past 14 years in our study area is indicative of the strong effect that the number of households has on local patterns of land use.

In the present case, most of these new households arise as a function of immigration, and not due to the establishment of new households by maturing young people in the villages. Many of these immigrants appear to be very similar to the “shifted cultivators” that have been previously described by Meyers [32]. Shifted cultivators are members of the community that move between rural areas in search of productive land for slash-and-burn agriculture. With the rapid depletion of soil nutrients and the consequent agricultural yield these shifted cultivators clear additional land until they are forced to move on in order to find fertile ground for cultivation. In Sambivinany and Sajateja, which lie along the primary road connecting the RNP region to Fionarantsoa, Antananarivo, and the coast, immigration rates are high and the productivity of agricultural plots are low. Both villages have the majority of their cultivated land in tavy, including 67% of the total area in tavy across the four study villages, with very little fallow land being reported. The result is a lower yield per area, which leads to less production than is likely necessary for subsistence. This low yield is apparently offset by ties to markets where households can purchase goods to offset low rice production. As a result, established households in these villages clear less forest land than established households in more remote villages. The higher deforestation rates associated with these villages are, instead, due to the establishment of new plots by immigrant households. Even those immigrant households near RNP that avoided tavy practices, presumably due to the diligent efforts of conservation organizations in the region, were more likely to establish sustainable paddy plots or family gardens (called tanimboly) than to engage in tavy. Clearly, the reduction in the use of swidden techniques associated with tavy farming (a practice that is now illegal in Madagascar) is important to preserve forest habitat and the biodiversity housed in it, but effective long-term strategies will need to account for the role of markets in the establishment and agricultural practices of migrant households.
